# Desmoplasia in cervical cancer is associated with a more aggressive tumor phenotype

**DOI:** 10.1038/s41598-023-46340-4

**Published:** 2023-11-02

**Authors:** Benjamin Wolf, Laura Weydandt, Nadja Dornhöfer, Grit Gesine Ruth Hiller, Anne Kathrin Höhn, Ivonne Nel, Rakesh K. Jain, Lars-Christian Horn, Bahriye Aktas

**Affiliations:** 1 Department of Gynecology, University Hospital Leipzig, Liebigstr. 20a, 04103 Leipzig, Germany; 2 Edwin L. Steele Laboratories, Department of Radiation Oncology, Massachusetts General Hospital, Boston, USA; 3 Harvard Medical School, Boston, USA; 4 Institute for Pathology, University Hospital Leipzig, Leipzig, Germany

**Keywords:** Cancer microenvironment, Gynaecological cancer, Tumour immunology

## Abstract

In cancer of the uterine cervix, the role of desmoplasia, i.e., peritumoral stromal remodeling characterized by fibroblast activation and increased extracellular matrix deposition, is not established. We conducted a retrospective cohort study based on data from 438 patients who had undergone surgical treatment for cervical cancer as part of the prospective Leipzig Mesometrial Resection study between 1999 and 2021. Using non-parametric tests, Kaplan–Meier plotting, and Cox regression modeling, we calculated the prognostic impact of desmoplasia and its association with other risk factors. Desmoplasia was present in 80.6% of cases and was associated with a higher frequency of lymphovascular space involvement (76.5 vs. 56.5%, *p* < 0.001) and venous infiltration (14.4 vs. 2.4%, *p* < 0.001). Lymph node metastasis (23.0 vs. 11.8%, *p* < 0.05) and parametrial involvement (47.3 vs. 17.6%, *p* < 0.0001) were also more common in patients with desmoplasia. The presence of desmoplasia was associated with inferior overall (80.2% vs. 94.5% hazard ratio [HR] 3.8 [95% CI 1.4–10.4], *p* = 0.002) and recurrence-free survival (75.3% vs. 87.3%, HR 2.3 [95% CI 1.2–4.6], *p* = 0.008). In addition, desmoplasia was associated with significantly less peritumoral inflammation (rho − 0.43, *p* < 0.0001). In summary, we link desmoplasia to a more aggressive phenotype of cervical cancer, reduced peritumoral inflammation, and inferior survival.

## Introduction

**Figure 1:**
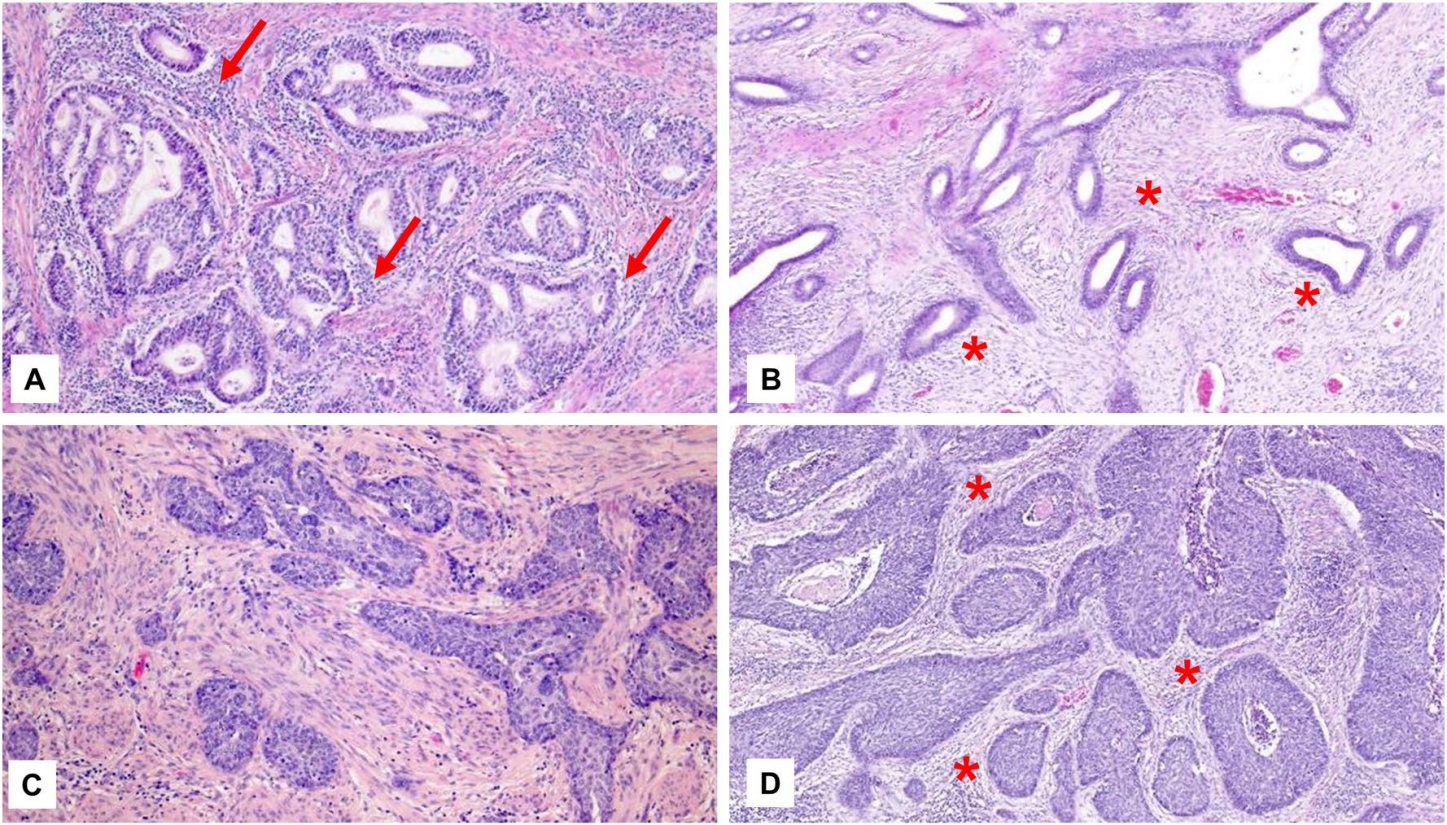
Histologic pictures of carcinomas of the uterine cervix, stained with hematoxylin–eosin. (**A**) adenocarcinoma of the uterine cervix without peritumoral desmoplastic change but weak peritumoral inflammatory response (arrow). (**B**) adenocarcinoma of the uterine cervix with strong immature peritumoral desmoplastic change, i.e., grade 3 desmoplasia (*). (**C**) squamous cell carcinoma of the uterine cervix with cell-rich stromal connective tissue but without peritumoral desmoplastic change. (**D**) squamous cell carcinoma of the uterine cervix with moderate peritumoral desmoplastic change (*).

While for many years cancer has been viewed as a disease of malignant cells, in recent decades the notion of cancer as a tissue level disorder has broadened the focus of research efforts to include the tumor microenvironment (TME) as an important player in promoting invasive growth and tumor progression^1^. Multiple studies have shown that changes in the TME cause transcriptional alterations in cancer cells leading to a more aggressive phenotype and promoting malignant progression^2–4^. Importantly, changes in the TME are not limited to structural alterations but also include peritumoral modifications of the immune response^5^. One readily observable type of TME alteration is the remodeling of peritumoral stroma by a switch from fibroblasts to myofibroblasts (i.e., cancer associated fibroblasts; CAF), extracellular matrix alterations (known as desmoplastic change), and neo-angiogenesis. In addition, a variable peritumoral inflammatory response can be observed. Desmoplasia has been observed in several tumors and it is a hallmark of some cancers such as pancreatic ductal adenocarcinoma^6^ or specific subtypes of medullary thyroid cancer^7^. Overall, desmoplastic remodeling of the tumor microenvironment has been shown to decrease tumor perfusion, cause hypoxia, and increase solid stress on pancreatic cancer cells. All these alterations promote proliferation, epithelial-mesenchymal transition (EMT), tissue invasion, and metastasis of malignant cells^8–12^. In addition, a more fibrotic tumor microenvironment can cause immunosuppression, characterized by reduced infiltration of cytotoxic lymphocytes into the tumor^13–15^. In this context, desmoplasia might both be a physical and a functional barrier for an efficient anti-tumor immune response. In gynecologic malignancies, and more specifically cervical cancer, desmoplasia has been described and shown to be associated with more aggressive tumor infiltration patterns^16–19^. Some research has also demonstrated inferior survival in patients with desmoplastic cervical cancer^19,20^. However, these studies were limited by small sample size, heterogenous outcome parameters, and only minimal inclusion of cases with advanced disease. That the TME plays an important and targetable role in cervical cancer treatment has been demonstrated by successful therapies with anti-VEGF treatment and immune-checkpoint blockade^21,22^. This report aims to dissect the role of desmoplasia in cervical cancer as one feature of peritumoral stromal alteration and discusses its implications for future therapies (Fig. 1).

## Results

**Figure 2:**
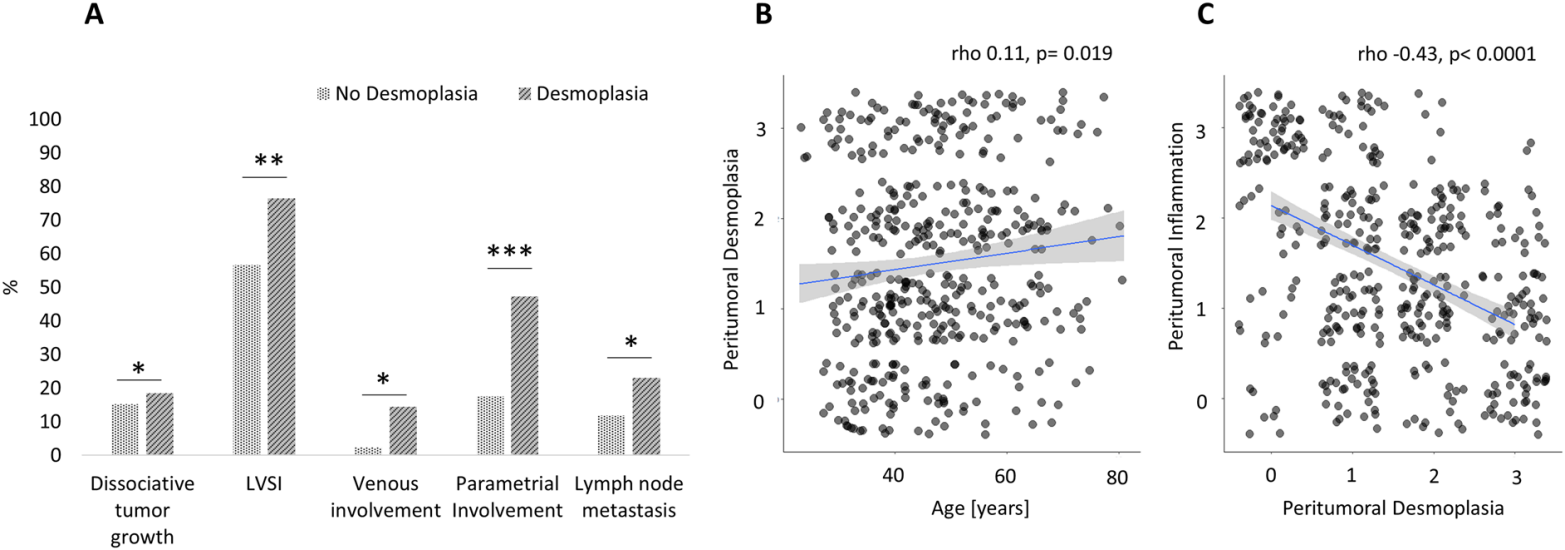
(**A**) Barplot showing the frequency of different risk factors in patients with desmoplastic and non-desmoplastic cervical cancer. *Indicates statistical significance of *p* < 0.05, **p < 0.001, ***p < 0.0001. LVSI, lymphovascular space invasion. (**B**) Scatterplot showing the linear correlation between patient age and peritumoral desmoplasia. For this comparison, the degree of desmoplasia was linearly transformed and represented on a scale from 0 to 3. Pearson’s r is given as a measure of correlation. (**C**) Scatterplot showing the inverse correlation between peritumoral desmoplasia and peritumoral inflammation. Both parameters were transformed into linear variables on the scale of 0–3. Pearson’s r is given as a measure of correlation.

**Figure 3:**
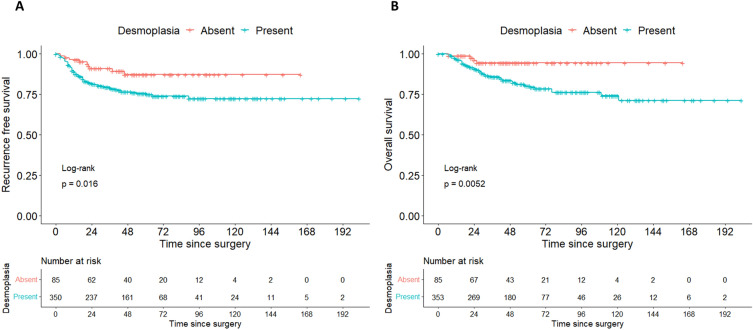
(**A**) Kaplan–Meier curves showing recurrence free survival of cervical cancer patients depending on the degree of desmoplasia. 5-year survival rates are given in Table 2. (**B**) Kaplan–Meier curves showing overall survival of cervical cancer patients depending on the degree of desmoplasia. 5-year survival rates are given in Table 2.

**Figure 4:**
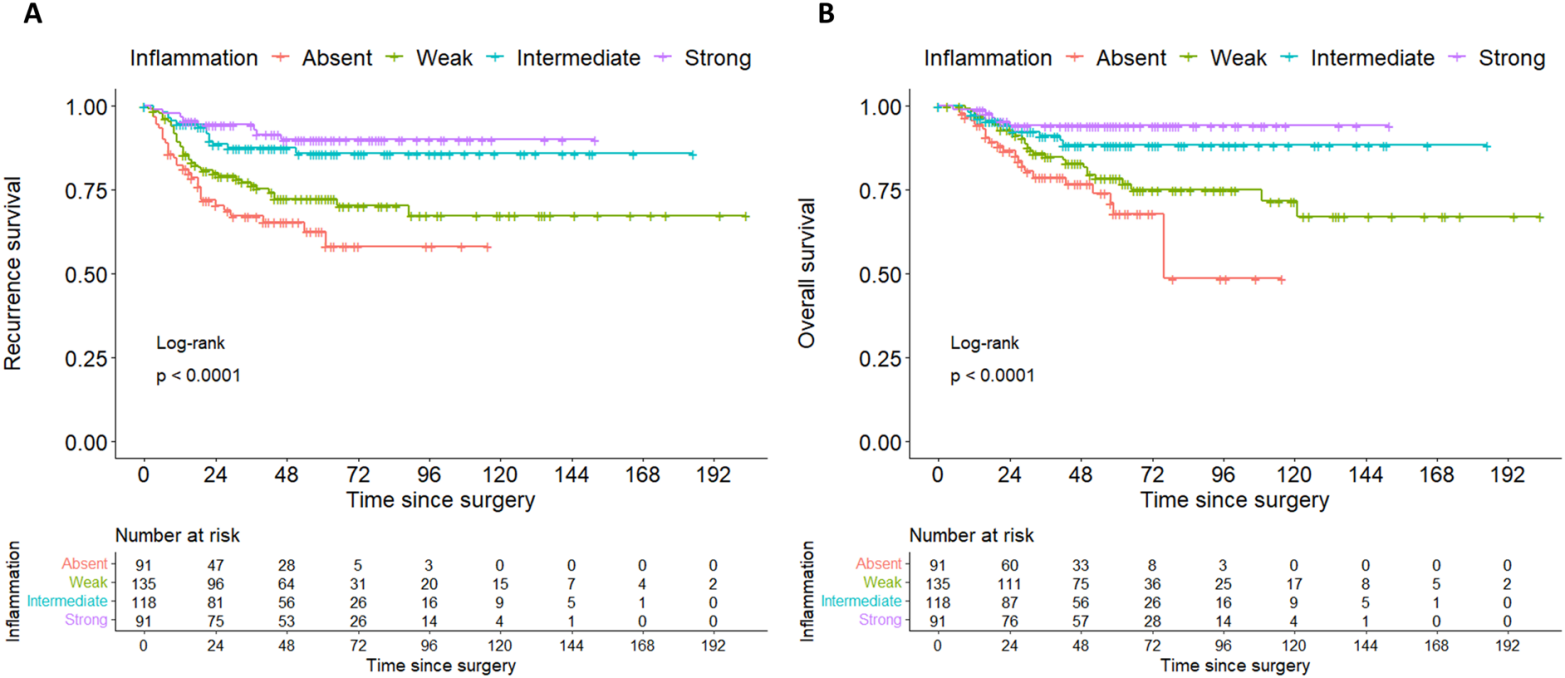
(**A**) Kaplan–Meier curves showing recurrence free survival of cervical cancer patients depending on the degree of peritumoral inflammation. (**B**) Kaplan–Meier curves showing overall survival of cervical cancer patients depending on the degree of peritumoral inflammation.

564 patients with primary cervical cancer treated surgically without any neoadjuvant therapy between 10/1999 and 01/2021 were identified in the MMR study database. The presence and/or extent of desmoplasia was not documented in the pathology reports of 126 patients, leaving 438 women to be included in this analysis. Desmoplasia was present in 353 (80.6%) patients. As observed in Table 1 and Fig. 2A, patients with desmoplasia had more frequently locally advanced carcinomas, lymphovascular and venous tumor infiltration, and pelvic and paraaortic lymph node metastasis. More advanced age was significantly associated with higher degrees of desmoplasia, although the degree of this correlation was small (rho 0.11, CI 0.02–0.2, *p* = 0.019, see Fig. 2B). There was an inverse correlation between the degree of desmoplasia and the degree of peritumoral inflammation (rho-0.43, CI − 0.5 to − 0.35, *p* < 0.0001, see Fig. 2C). Median follow-up time was 58 months (IQR 32–74 months). Both the recurrence free (Fig. 3A) and overall survival (Fig. 3B) were significantly inferior in patients with desmoplasia, with higher degrees of desmoplasia being associated with successively inferior survival rates (Table 2, Figure S1). Because recurrence free and overall survival showed equal patterns over time, we restricted subsequent subgroup analyses to overall survival, which we considered to be the more definite endpoint. The degree of peritumoral inflammation was directly related to the probability of survival with higher degrees of inflammation being associated with better survival (see Fig. 4A,B).

**Table 1:** Clinical and pathological patient data.

		No desmoplasia n = 85		Desmoplasia n = 353		*p* value
		N	%	n	%	
Age in years (IQR)		40 (34–50)		47 (39–57)		0.001^a^
BMI kg/m^2^ (IQR)		24 (22–27)		24 (22–27)		0.82^a^
Tumor size in mm (IQR)		32 (22–42)		37 (27–49)		0.02^a^
FIGO stage (preoperative)	IB1	41	48.2	148	41.9	0.009^b^
	IB2	16	18.8	29	8.2	
	IIA1	5	5.9	10	2.8	
	IIA2	0	0.0	10	2.8	
	IIB	21	24.7	134	38.0	
	IIIA	0	0.0	2	0.6	
	IIIB	2	2.4	13	3.7	
	IVA	0	0.0	7	2.0	
pT stage (postoperative)	1b1	48	56.5	130	36.8	< 0.0001^b^
	1b2	17	20.0	39	11.0	
	2a1	3	3.5	11	3.1	
	2a2	2	2.4	6	1.7	
	2b	15	17.6	157	44.5	
	3b	0	0.0	2	0.6	
	4	0	0.0	8	2.3	
pN status	N0	75	88.2	272	77.1	0.02^c^
	N1	10	11.8	81	23.1	
pM (LYM) status	M0	22	25.9	109	30.9	0.02^b^
	M1	0	0.0	32	9.1	
	not assessed	63	74.1	212	60.1	
Grading	1	17	20.0	50	14.2	0.25^b^
	2	30	35.3	154	43.6	
	3	38	44.7	149	42.2	
Lymphovascular space involvement	absent	37	43.53	83	23.5	0.0002^c^
	present	48	56.5	270	76.5	
Venous infiltration	absent	83	97.6	302	85.6	0.002c
	present	2	2.4	51	14.4	
Histological subtype	Squamous cell carcinoma	64	75.3	271	76.8	0.9^b^
	Adenocarcinoma	18	21.2	67	19.0	
	Adenosquamous	3	3.5	13	3.7	
	Other	0	0.0	2	0.6	
Recurrence	None	76	89.4	273	77.3	0.04^c^
	Pelvic	4	4.7	31	8.8	
	Distant (+ /− pelvic)	5	5.9	49	13.9	

^a^Mann-Whitney-U test, ^b^exact Fisher test, ^c^chi-squared test.

**Table 2:** Survival data.

Overall survival		n	5-year OAS (%)	95% CI	*p* value	Median follow-up in months (IQR)
All patients (n = 438)		438	82.8	78.8–87.0		58 (32–74)
All patients	No Desmoplasia	85	94.5	89.3–99.9	0.015	
	Weak desmoplasia	141	82.1	75.0–89.8		
	Intermediate desmoplasia	119	80.6	72.7–89.4		
	Strong desmoplasia	93	76.8	67.8–87.0		
pN0	No Desmoplasia		98.4	95.2–100	0.028	
	Desmoplasia		88.6	84.6–93.4		
pN1	No desmoplasia		63.0	36.3–100	0.65	
	Desmoplasia		50.0	39.3–63.6		

Recurrence free survival		n	5-year RFS	95% CI	*p* value	Median follow-up in months (IQR)
All patients (n = 438)		438	77.6	73.5–82.0		57 (31–73)
All patients	No Desmoplasia	85	87.3	79.7–95.6	0.037	
	Weak desmoplasia	141	78.5	71.5–86.2		
	Intermediate desmoplasia	119	73.0	64.5–87.7		
	Strong desmoplasia	93	73.0	64.0–83.2		
pN0	No Desmoplasia	75	90.1	82.7–98.1	0.12	
	Desmoplasia	272	83.6	79.0–88.6		
pN1	No desmoplasia	10	60.0	32.9–100	0.34	
	Desmoplasia	81	47.0	35.3–62.5		

CI: confidence interval. Statistical significance was determined by logrank tests.

Out of 91 cases with lymph node metastasis, only 8 (8.7%) had no evidence of peritumoral desmoplasia. As expected, the presence of desmoplasia and other markers of invasiveness had therefore no prognostic impact in these patients. On the other hand, desmoplasia was able to stratify node-negative patients into high-risk and low-risk subsets (Figure S2). Desmoplasia had the same impact on patients with adenocarcinoma or squamous cell carcinoma (Figure S3). We found that the absence of desmoplasia identified subgroups of patients with considerably lower risk among women with high-grade tumors (Figure S4) and among women whose tumors exhibited lymphovascular space invasion (Figure S5).

Results from univariable Cox proportional hazard regression modeling using age, tumor size, LVSI, grading, histological subtype, desmoplasia, parametrial infiltration, and lymph node status are presented in Table 3. The presence of desmoplasia was associated with an increased risk for death (HR 3.8, CI 1.4–10.4, *p* < 0.01). Using desmoplasia as continuous variable ranging from 0 to 3, univariate regression revealed significance with a HR of 1.5 per desmoplasia grade (CI 1.1–1.8, *p* < 0.01).

**Table 3:** Regression analysis.

	Factor	HR	95% CI	*p* value
Univariable				
Continous	Age (years)	1.01	0.99–1.03	0.2
	Tumor size (mm)	1.023	1.02–1.03	< **0**.**0001**
	Desmoplasia (0–3)	1.3	1.09–1.64	< **0**.**01**
Binomial	Grading (G3 vs. G1/G2)	2.5	1.6–3.8	< **0**.**0001**
	LVSI (L1 vs. L0)	12.4	3.92–39.16	< **0**.**0001**
	Histological type (adeno vs. squamous cell carcinoma)	0.98	0.57–1.67	0.94
	Desmoplasia (present vs. absent)	2.3	1.2–4.6	**0**.**02**
	Parametrial infiltration (present vs. absent)	5.9	3.6–9.7	< **0**.**0001**
	Lymph node metastasis (pN1 vs. pN0)	5.4	3.5–8.2	< **0**.**0001**
Multivariable				
Continuous	Tumor size (mm)	1.013	1.002	**0**.**02**
Binomial	Grading (G3 vs. G1/G2)	1.47	0.95–2.28	0.08
	LVSI (L1 vs. L0)	5.33	1.62–17.52	< **0**.**01**
	Desmoplasia (present vs. absent)	1.17	0.57–2.4	0.67
	Parametrial infiltration (present vs. absent)	2.46	1.4–4.3	< **0**.**01**
	Lymph node metastasis (pN1 vs. pN0)	2.2	1.4–3.4	**0**.**001**

LVSI, lymphovascular space invasion; CI, confidence interval; *P* values were determined by logrank tests.

Significant values are in [bold].

In a multivariable Cox-regression model including all parameters significant on univariable regression, tumor size, lymphovascular space invasion (LVSI), parametrial invasion, and lymph node metastasis remained significant predictors of death from any cause (see Table 3).

Because we found that desmoplasia has a differential prognostic impact in patients with and without lymph node metastasis (Figure S2), we compared the role of desmoplasia in a multivariable context in both cohorts (pN0 and pN1). Even though desmoplasia had a larger prognostic effect in patients without pelvic and/or paraaortic lymph node metastasis (HR 3.9, CI 0.52–29.7) compared to patients with lymph node metastasis (HR 0.75, CI 0.21–2.7) this effect was not significant in either model (Figure S6). We could therefore not establish desmoplasia as an independent prognostic marker, either as a binomial or as a continuous parameter.

## Discussion

In this study, we demonstrate that peritumoral desmoplastic remodeling of the stroma occurs in most, but not all carcinomas of the uterine cervix, regardless of histologic subtype. When desmoplasia is present, it is associated with a more aggressive tumor phenotype indicated by lymphovascular space invasion, venous infiltration, lymph node metastasis, and parametrial invasion. These characteristics translate into significantly inferior recurrence-free and overall survival as compared to patients without desmoplasia. Nevertheless, it should be emphasized that the aim of this investigation was to advance our understanding on the biology and morphology of the cervical cancer microenvironment rather than provide immediate diagnostic or therapeutic recommendations. However, some diagnostic and therapeutic implications will be discussed below.

Prior retrospective studies have reported similar results on smaller and highly selected cohorts: Akimoto and colleagues examined the role of desmoplasia in 59 early stage cervical adenocarcinoma patients^20^. In line with our results, they reported that the presence of desmoplasia was inversely related to peritumoral inflammation, and that tumors exhibiting mature desmoplasia (i.e., no or minimal desmoplasia according to the terminology used in our paper) had a significantly better prognosis than tumors with immature desmoplasia (i.e., grade 3 desmoplasia). As in our study, the presence of desmoplasia was associated with lymphovascular space invasion, lymph node metastasis, and parametrial infiltration, however, no multivariable analyses were performed. In another study including 20 cases of cervical adenocarcinoma in situ (AIS) or early invasive adenocarcinoma, the authors demonstrated that the expression of α-smooth muscle actin (α-SMA) was the most sensitive marker of invasion compared to six other surface antigens, indicating that desmoplasia is a reliable feature of invasiveness^23^. No oncologic outcomes were reported in that investigation. Our study included 85 AC cases staged pT1b1 or larger with 27 (32%) exhibiting parametrial involvement and 15 (17.6%) having lymph node metastasis. We can therefore extend the previous findings to more advanced cases in a larger cohort.

Other investigators have also noted significant peritumoral tissue changes characterized by the loss of CD34 and a gain of α-SMA expression in the stroma surrounding cervical cancers and pre-cancerous lesions^24–26^. Cao et al. analyzed 122 squamous cell cervical cancer cases and found that desmoplasia was independently associated with worse distant-metastasis free survival^19^. In addition, the investigators demonstrated that tumor cell budding—a phenomenon indicative of dissociative tumor growth and a more aggressive phenotype—was associated with desmoplasia, in line with the findings from our study and Horn et al.^16^. The study reported very limited clinical information and restricted its outcome analysis to distant metastasis (lungs, supraclavicular lymph nodes, and bones)^19^.

As desmoplasia is not an independent prognostic marker, but rather a phenomenon associated with known risk factors for adverse outcomes, why should it be of interest?

First, as other investigators have demonstrated, desmoplasia is present already in early disease^23^, preceding other manifestations of cancer progression such as lymphovascular space invasion. As desmoplasia is more uniformly present throughout a tumor, it might be easier to identify this feature in smaller tissue samples compared to other histopathological risk features such as LVSI.

Interestingly, it has been shown in preclinical models that a stiff collagen matrix—the hallmark of desmoplasia—facilitates EMT^12^ as well as cancer cell invasion into the surrounding tissue including the vascular system by a process termed cell streaming migration^27,28^.

Cervical cancers that can reprogram tissue resident fibroblasts into CAFs to produce a desmoplastic tumor microenvironment might therefore constitute a particularly aggressive subtype of this disease from the beginning, while tumors which do not possess this ability follow a more indolent course without lymphovascular space invasion and lymph node metastasis.

Second, there is increasing evidence that desmoplasia is a *targetable* mechanism of tumor progression. The desmoplastic, fibrotic stroma shields the tumor from both the physiologic immune response mounted against a neoplasm, and the influence of therapeutic substances such chemotherapeutic agents. This mechanism is related to local blood vessel compression as has been elegantly shown in multiple experiments^29–31^. Therefore, treatments targeting CAFs subsequently leading to a reversal of peritumoral desmoplasia would be desirable. One promising approach is targeting the angiotensin signaling pathway. Studies conducted with pancreatic cancer models have demonstrated that (i) losartan (an angiotensin receptor blocker) reprograms pro-fibrogenic CAFs, (ii) decreases extracellular matrix, (iii) reduces abnormal extracellular forces, (iv) enhances therapeutic efficacy by improving drug delivery in orthotopic tumor models, and (v) reprograms the immunosuppressive TME into an immunostimulatory milieu^32–35^. Recently, exciting results have been published showing that the addition of losartan to standard neoadjuvant treatment increases the R0 resection rates and improves survival in pancreatic cancer patients^36^. In addition, a recent retrospective study involving more than 10,000 cancer patients treated with immune checkpoint inhibitors showed that overall survival was improved in those patients taking angiotensin inhibitors, especially in patients with gastrointestinal (GI) or genitourinary (GU) cancers^37^.

Reducing desmoplasia pharmacologically could lead to a stronger peritumoral immune response resulting in better tumor control, effectively turning cold tumors hot. Especially in patients with advanced disease who now frequently receive immune checkpoint inhibitors, such a reprogramming of the tumor microenvironment might be beneficial. In addition, there is great interest in developing chimeric antigen receptor (CAR) cell therapies for patients with solid tumors including cervical cancer, an undertaking that has been difficult, among other reasons because of the complex tumor microenvironment^38,39^. Removing some barriers by reducing desmoplasia might be a promising approach. Our findings establish the critical role of desmoplasia in cervical cancer, paving the way for future studies in this direction. In the case of thyroid medullary cancer, evaluation for the presence of peritumoral desmoplasia can aid the decision whether lymph node dissection should be performed or not^40^.

Our study has important limitations: first, it is a monocentric, retrospective analysis of data gathered as part of a surgical trial. Because no information on desmoplasia was available in 126 patients, this led to a significant reduction of our study cohort, potentially introducing some bias. Importantly, most patients who were excluded were recruited early during the study period as desmoplasia was not routinely assessed during these years. This also led to a significant reduction in our follow-up time. Second, our study cohort only involved surgically treated patients, excluding patients who received chemo-radiotherapeutic treatment which is the standard of care for advanced disease at most centers^41^. Our findings are therefore not comparable to other cohorts. However, we would expect the prognostic impact of desmoplasia to be even more pronounced in patients undergoing chemo-radiotherapy, as the delivery of chemotherapeutic and radio-sensitizing agents (usually cisplatin) would be expected to be hindered by the presence of desmoplasia. The same could be expected for patients with metastatic disease undergoing primary chemotherapy involving treatment with the anti-VEGF antibody bevacizumab or with an immune checkpoint inhibitor as outlined above. Finally, we excluded patients with locally advanced disease involving the distal vagina, the pelvic side wall, or adjacent organs such as the urinary bladder or rectum (i.e., stage IIIA–IVA), as these patients are only rarely treated by surgery. Exclusion of these cases limits the generalizability of our findings but considering the small number of cases excluded (n = 7), we do not think that this affected our results.

On the other hand, our study is unique involving a large number of surgically treated patients, many with advanced disease. Additionally, the degree of desmoplastic change as well as of peritumoral inflammatory response were evaluated during routine workup of the surgical specimens, highlighting its practical applicability.

### Conclusion

In summary, we show that desmoplastic stromal remodeling is associated with a more aggressive phenotype of cervical cancer, reduced immunocyte infiltration, and inferior survival. Targeting desmoplasia should be considered as a potential treatment approach.

## Methods

### Patient selection and data acquisition

This is a retrospective sub-group analysis from the prospective, monocentric, observational Mesometrial Resection (MMR) study at our institution which was initiated in September 1999 to evaluate a novel surgical strategy for the treatment of cervical cancer based on the theory of ontogenetic cancer fields. The study was approved by the Leipzig University Ethics Committee (151/2000, 192/2001, and 012/13–28012013. Initial approval was granted on 22 September 2000, and the subsequent amendments were approved on 17 October 2007 and 6 March 2013). The study was registered retrospectively at the German Clinical Trials Registry (DRKS00015171). All procedures and methods in the trial were performed in accordance with the approved protocols as well as the Declaration of Helsinki and relevant local and national guidelines and regulations. A detailed description of the trial has been published along with its results^42^ and is available at https://www.drks.de/drks_web/setLocale_EN.do. All patients provided informed consent to participate in this study which included the permission to use data for further analysis.

For this analysis, we searched our MMR study database to identify patients with cervical cancer staged IB1 to IVA according to the 2009 version of the Fédération internationale de gynécologie et d'obstétrique (FIGO) who had undergone primary surgical resection at the University Hospital Leipzig between 10/1999 and 12/2021. Besides the exclusion criteria specified in the MMR study protocol such as previous major pelvic surgery and the presence of severe systemic disease prohibiting surgery (American Society of Anesthesiologists [ASA] score ≥ 3), for this current analysis we also excluded women who had undergone neoadjuvant treatment with chemotherapy or had had radiotherapy to the pelvis in the past. Although permitted in the MMR trial, patients who had undergone simple hysterectomy previously were excluded from the analysis presented here. All surgical specimens had been assessed postoperatively by a gynecological pathologist. The presence and extent of peritumoral desmoplasia and inflammation were specified in most pathology reports but not primarily collected in our study database. This information was therefore gathered retrospectively. Patients for whom this information was not available were excluded from the present analysis. Patients were followed-up in person at our outpatient clinic every 3 months during the first two years and every 6 months until completion of the 5th postoperative year. Patients wishing further follow-up at our hospital were seen once a year thereafter. Other patients were contacted by phone every 2–3 years to confirm survival and recurrence status.

### Histological assessment of desmoplasia and peritumoral inflammation

The intensities of peritumoral desmoplastic reaction, tumor growth pattern, and peritumoral inflammation were determined in a microscopic field using a tenfold objective and routine hematoxylin–eosin (HE) staining as described before^16–18^. Histologically, stromal desmoplasia is characterized by bands of slim undulating stromal cells within abundant collagen. We applied a semiquantitative score ranging from absent (0) to low (1), moderate (2), and strong (3) for peritumoral desmoplasia and likewise for peritumoral inflammation (see Fig. 1).

### Statistical analysis

All data were recorded in Excel spreadsheets (Microsoft, 2016) and comma-separated-variable (CSV) tables were created for further statistical processing in *R*, an open-source statistical software^43^. Continuous data are presented in medians and inter-quartile ranges (IQR) while categorical data are given as percentages. Confidence intervals (CI), when applicable, are given for the 95% range. Built-in parametric tests were used to calculate the significance of differences between groups (i.e., Fisher’s exact test, chi-squared test, and the Mann–Whitney-U test). For a more robust correlation between peritumoral inflammation and desmoplasia, (both continuous processes by nature), we transformed both parameters into linear variables reaching from 0 to 3. We then calculated Pearson’s product moment correlation using the built-in R function *cor.test*. Observed differences between groups were considered statistically significant if the *p* value was less than 0.05.

Recurrence free survival was defined between the date of operation and radiologically or histologically proven disease relapse. Patients who died without evidence of recurrence were censored at the time of death. Overall survival was defined as the time from surgical treatment to death from any cause. Survival estimates were calculated using the Kaplan–Meier analysis in the survival package for R^44^. The *coxph* function was used to build the Cox proportional hazard models. Survival curves and forest plots were drawn using the ggplot2^45^ and survminer^46^ packages. Median follow-up times were calculated as the inverse Kaplan–Meier function of the respective survival curves. Differences in survival between groups were quantified using hazard ratios from Cox regression modeling. The logrank test implemented in the survival package was used to calculate the statistical significance of differences between survival curves.

### Ethical approval

The study was approved by the Leipzig University Ethics Committee (151/2000, 192/2001, and 012/13–28012013; initial approval was granted on 22 September 2000, and the subsequent amendments were approved on 17 October 2007 and 6 March 2013).

### Supplementary Information

Supplementary Information.

## Data Availability

We will provide our data for independent analysis by qualified investigators or for the reproducibility of this study in other centers if such a request is made to the corresponding author.
