# Supplementary material for: Desmoplasia in cervical cancer is associated with a more aggressive tumor phenotype
Source: Sci Rep. 2023 Nov 2;13:18946. doi: 10.1038/s41598-023-46340-4 (PMC10622496; doi:10.1038/s41598-023-46340-4)
Supplement: Supplementary file 1 — Supplementary Information. [file 41598_2023_46340_MOESM1_ESM.pdf]

Supplementary appendix

Desmoplasia in cervical cancer is associated with a more aggressive tumor phenotype

Benjamin Wolf, Laura Weydandt, Nadja Dornhöfer, Grit Gesine Ruth Hiller, Anne Katrin Höhn, Ivonne Nel, Rakesh K. Jain, Lars-Christian Horn, and Bahriye Aktas

Tab. S1:

**Overall survival**		**n**	**5-year OAS (%)**	**95% CI**	**p-value**	**Median follow-up in months (IQR)**
All patients (n=438)		438	82.8	78.8-87.0		58 (32-74)
All patients	No Desmoplasia	85	94.5	89.3-99.9	0.015	
	Weak desmoplasia	141	82.1	75.0-89.8		
	Intermediate desmoplasia	119	80.6	72.7-89.4		
	Strong desmoplasia	93	76.8	67.8-87.0		
pN0	No Desmoplasia		98.4	95.2-100	0.028	
	Desmoplasia		88.6	84.6-93.4		
pN1	No desmoplasia		63.0	36.3-100	0.65	
	Desmoplasia		50.0	39.3-63.6		

**Recurrence free survival**		**n**	**5-year RFS**	**95% CI**	**p-value**	**Median follow-up in months (IQR)**
All patients (n=438)		438	77.6	73.5-82.0		57 (31 - 73)
All patients	No Desmoplasia	85	87.3	79.7-95.6	0.037	
	Weak desmoplasia	141	78.5	71.5-86.2		
	Intermediate desmoplasia	119	73.0	64.5-87.7		
	Strong desmoplasia	93	73.0	64.0-83.2		
pN0	No Desmoplasia	75	90.1	82.7-98.1	0.12	
	Desmoplasia	272	83.6	79.0-88.6		
pN1	No desmoplasia	10	60.0	32.9-100	0.34	
	Desmoplasia	81	47.0	35.3-62.5		

CI: confidence interval

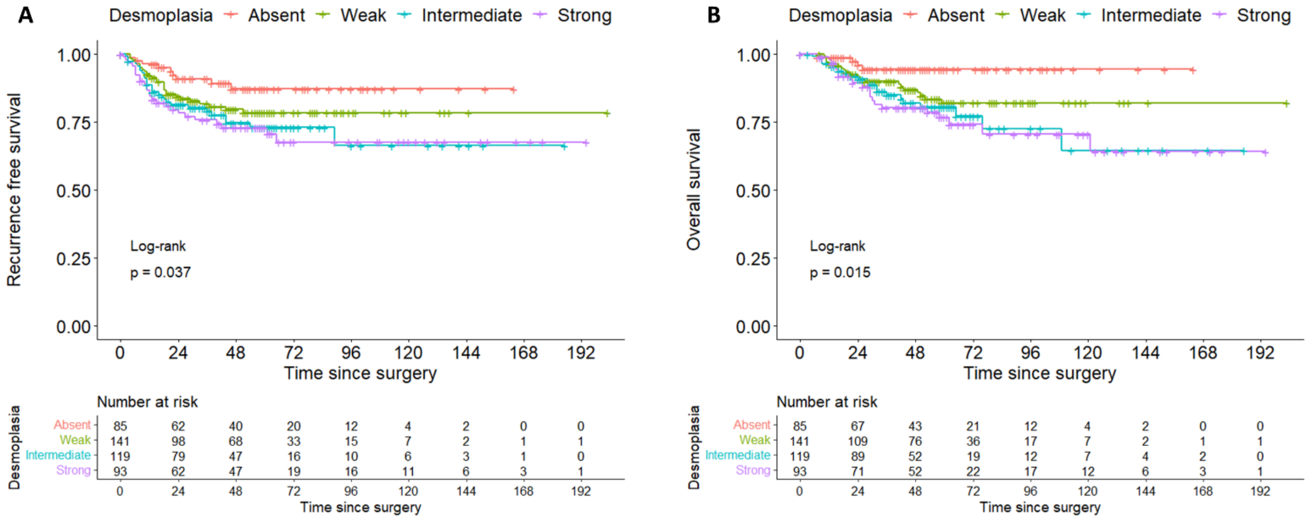

Fig. S1: Recurrence free **(A)** and overall **(B)** survival probabilities as a function of degree of desmoplasia. Refer to table 2 for pertinent data.

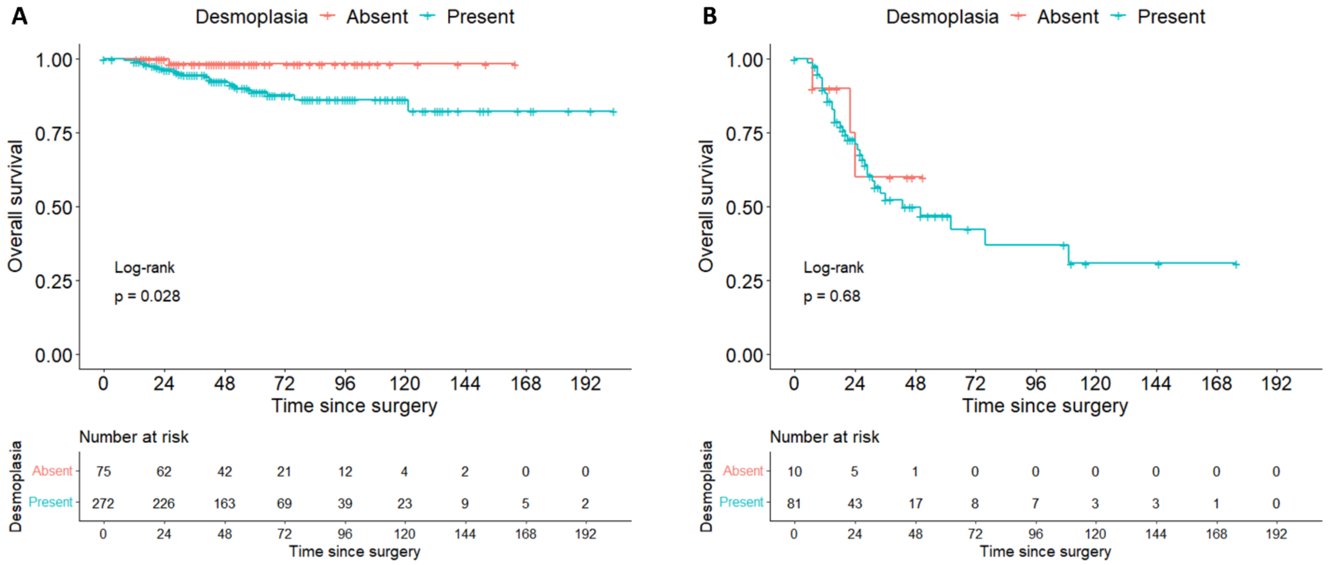

Fig. S2: Overall survival in patients without lymph node metastasis **(A)** and with lymph node metastasis **(B)** stratified for the presence or absence of desmoplasia.

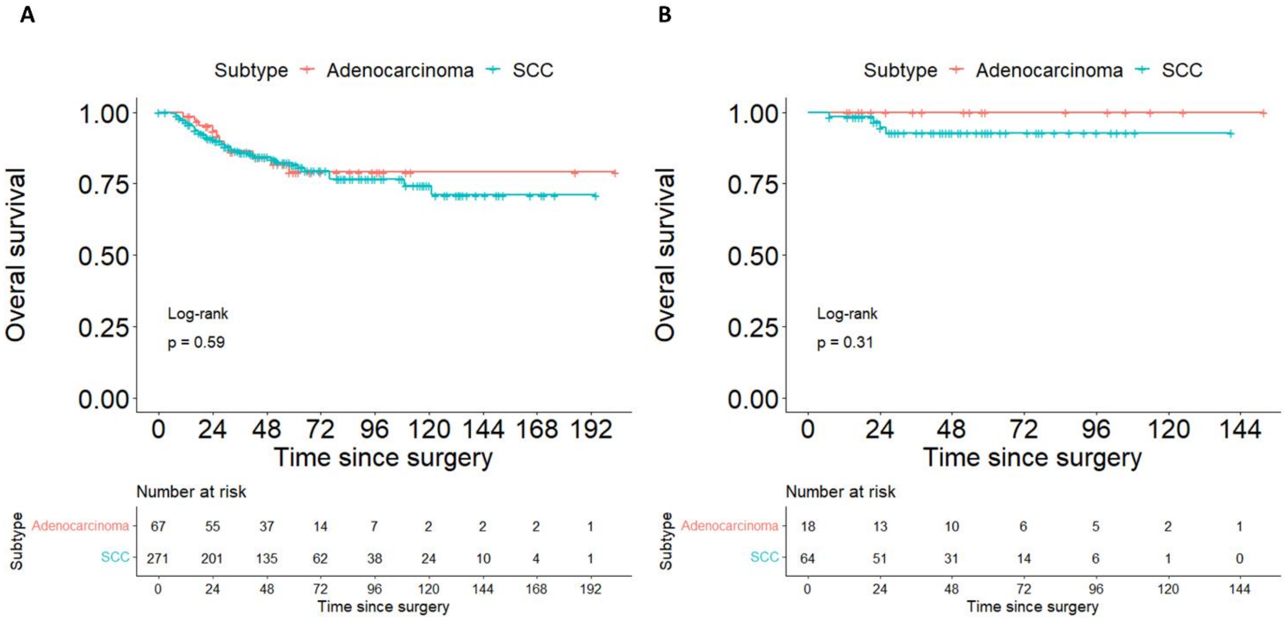

Fig. S3: Overall survival in patients with desmoplasia **(A)** and without desmoplasia **(B)** depending on histological subtype. Patients with adenosquamous or other histological subtypes (n=18, 3 without desmoplasia and 15 with demoplasia) were excluded from this analysis.

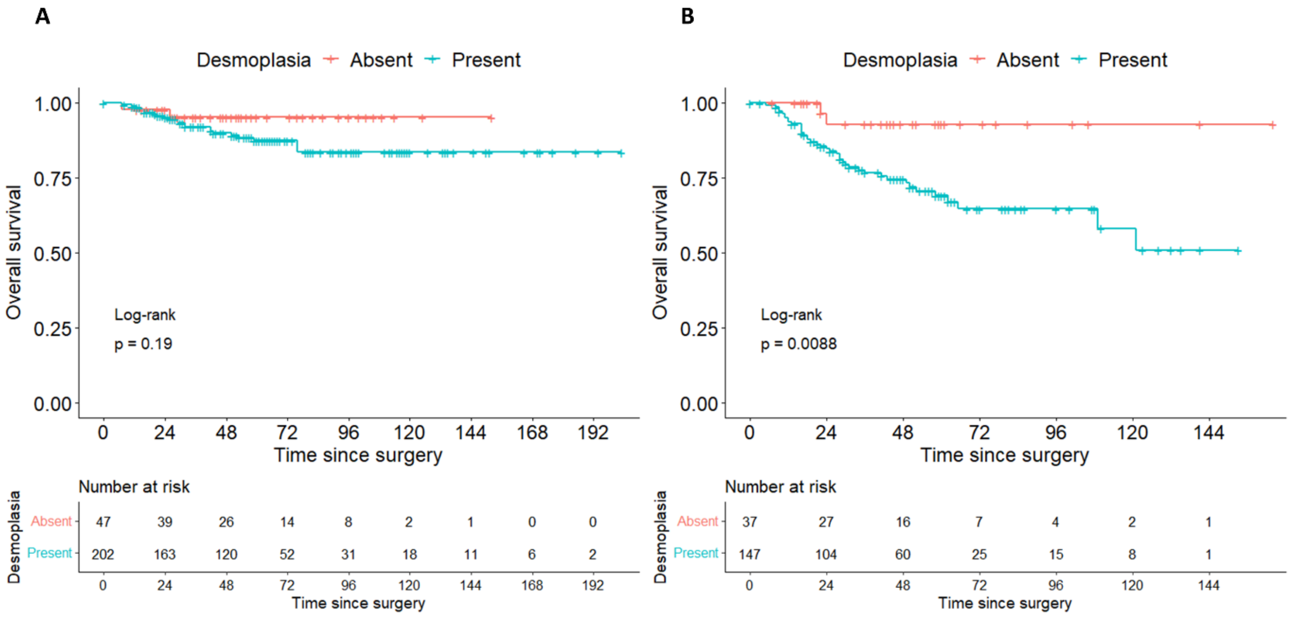

Fig. S4: Overall survival in patients with low grade (i.e., G1 and G2) tumors **(A)** and high grade (i.e., G3) tumors **(B)** depending on whether desmoplasia was present or not.

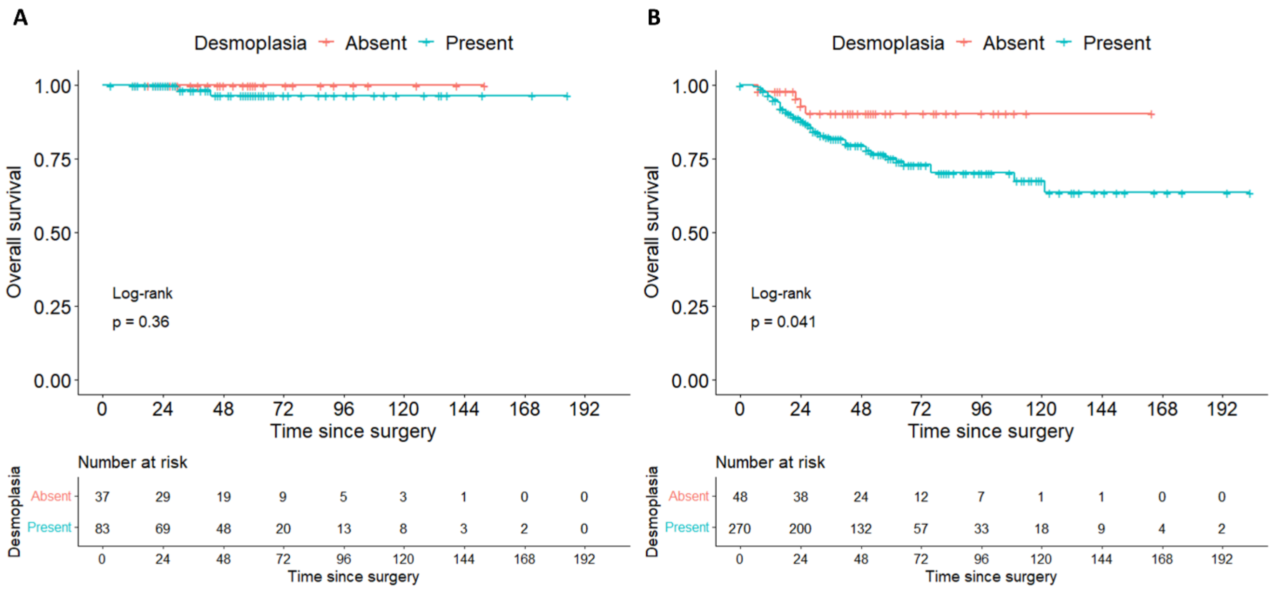

Fig. S5: Overall survival in patients with lymphovascular space invasion **(A)** and without lymphovascular space invasion **(B)** depending on the presence or absence of desmoplasia.

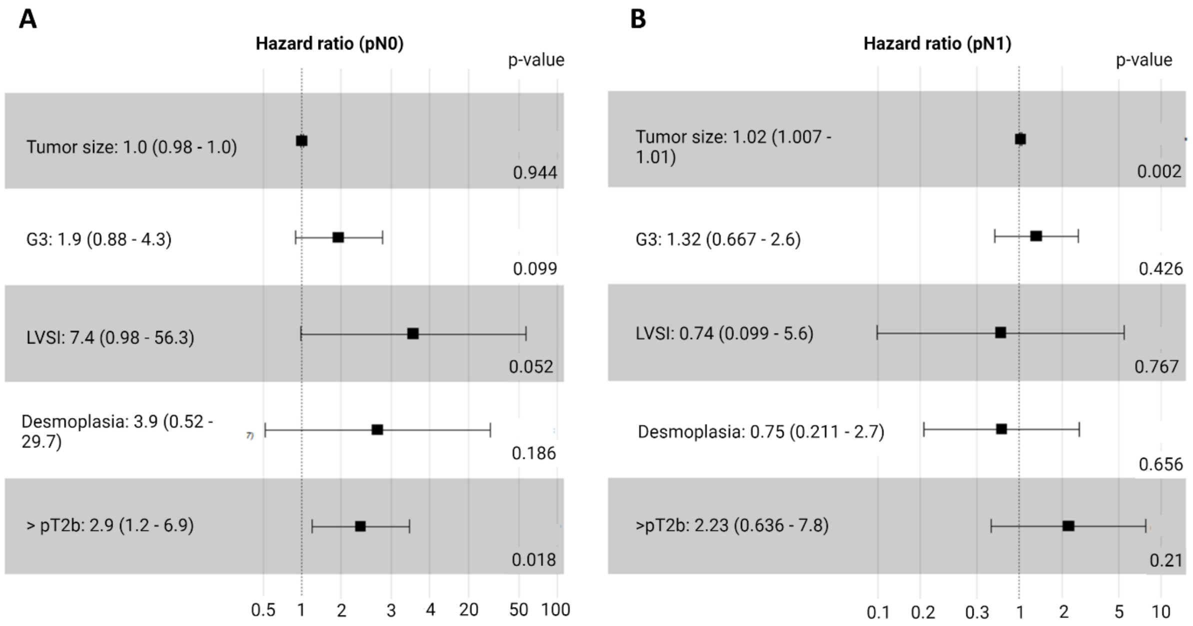

Fig. S6: Forest plots of multivariable Cox regression models showing the hazard ratios of common risk factors in patients without lymph node metastasis (pN0, **A**) and with lymph node metastasis (pN1, **B**). Also refer to table 3 in the main text.
